# MoTe_2_ Photodetector for Integrated Lithium Niobate Photonics

**DOI:** 10.3390/nano15010072

**Published:** 2025-01-05

**Authors:** Qiaonan Dong, Xinxing Sun, Lang Gao, Yong Zheng, Rongbo Wu, Ya Cheng

**Affiliations:** 1 State Key Laboratory of High Field Laser Physics and CAS Center for Excellence in Ultra-Intense Laser Science, Shanghai Institute of Optics and Fine Mechanics (SIOM), Chinese Academy of Sciences (CAS), Shanghai 201800, China; dongqn@shanghaitech.edu.cn (Q.D.); langgao@siom.ac.cn (L.G.); 2 School of Physical Science and Technology, ShanghaiTech University, Shanghai 200031, China; 3 The Extreme Optoelectromechanics Laboratory (XXL), School of Physics and Electronic Science, East China Normal University, Shanghai 200241, China; yong.zheng@stu.ecnu.edu.cn (Y.Z.); rbwu@phy.ecnu.edu.cn (R.W.)

**Keywords:** on-chip photodetectors, MoTe_2_, microresonator, lithium niobate photonics, photolithography-assisted chemical–mechanical etching

## Abstract

The integration of a photodetector that converts optical signals into electrical signals is essential for scalable integrated lithium niobate photonics. Two-dimensional materials provide a potential high-efficiency on-chip detection capability. Here, we demonstrate an efficient on-chip photodetector based on a few layers of MoTe_2_ on a thin film lithium niobate waveguide and integrate it with a microresonator operating in an optical telecommunication band. The lithium-niobate-on-insulator waveguides and micro-ring resonator are fabricated using the femtosecond laser photolithography-assisted chemical–mechanical etching method. The lithium niobate waveguide-integrated MoTe_2_ presents an absorption coefficient of 72% and a transmission loss of 0.27 dB µm^−1^ at 1550 nm. The on-chip photodetector exhibits a responsivity of 1 mA W^−1^ at a bias voltage of 20 V, a low dark current of 1.6 nA, and a photo–dark current ratio of 10^8^ W^−1^. Due to effective waveguide coupling and interaction with MoTe_2_, the generated photocurrent is approximately 160 times higher than that of free-space light irradiation. Furthermore, we demonstrate a wavelength-selective photonic device by integrating the photodetector and micro-ring resonator with a quality factor of 10^4^ on the same chip, suggesting potential applications in the field of on-chip spectrometers and biosensors.

## 1. Introduction

On-chip photonic integrated circuits (PICs), due to their ultrahigh processing speeds and low power consumption, have gained extensive attention in recent years for their advantages in data processing, communication, and various sensing applications [1]. The major PIC platforms progressing toward industrial-scale development include silicon-on-insulator (SOI) [2], silicon nitride (Si_3_N_4_) [3], indium phosphide (InP) [4], and lithium-niobate-on-insulator (LNOI) [5]. Among these materials, LiNbO_3_ (LN) exhibits numerous beneficial properties, such as a wide transparent window, strong second-order nonlinearity, the capability for periodic polarization, and fast, low-loss switching through the electro-optic effect [6].

In the past few years, the commercialization of lithium niobate on insulator substrates has made it possible to achieve large-scale fabrication of high-density integrated, compact, and low-loss photonic integrated circuit components [7]. Thanks to the fabrication of low-loss LNOI waveguides using femtosecond laser-assisted photochemical etching [8], researchers have extensively explored the integration of photonic components on the LNOI platform, including the electro-optic modulators [9], optical frequency combs [10], delay lines [11], lasers [12], amplifiers [13], and acousto-optic modulators [14]. The integration of photodetectors (PDs) is crucial for scalable integrated lithium niobate photonics since free-space coupling into photodetectors invariably introduces coupling losses, thereby lowering the overall system efficiency. Moreover, heterogeneous integration provides a direct pathway to improve the functionality, reliability, and scalability of these devices. However, on-chip photodetectors operating at telecom-band wavelengths remain a continual challenge, which is mostly solved by hetero-integrating bulk III-V compounds and germanium semiconductors [15,16].

Thanks to two-dimensional (2D) materials being bonded by weak van der Waals forces, they could be exfoliated and stacked arbitrarily (not limited by lattice matching). A promising direction under exploration involves the use of 2D materials as active layer materials for photodetectors [17]. Typically, 2D transition metal dichalcogenide (TMDC) materials exhibit exceptional properties, including ultrahigh photoresponsivity [18], broadband photodetection [19], ultralow dark current [20], and substantial photocurrent on/off ratios, when compared to gapless graphene and low-stability black phosphorus [21]. However, most of the TMDC (i.e., MoS_2_, MoSe_2_, and WS_2_) photodetectors operate in the visible wavelength range due to their wide bandgap [22,23,24]. As a member of the TMDCs, 2H-MoTe_2_ exhibits the variable electronic properties, mechanical flexibility, and optical sensitivity characteristic of this group [21], while its small bandgap of 0.93 to 1.05 eV with layer-dependent features makes it an excellent candidate for near-infrared optoelectronic applications [25]. Recently, a high-speed on-chip photodetector employing a few-layer 2H-MoTe_2_ onto a silicon waveguide operating in the O-band telecommunication spectral range was experimentally demonstrated by Ma et al. [26]. Chen Li et al. demonstrated 2H-MoTe_2_ p-i-n homojunctions fabricated directly on a silicon photonic crystal waveguide with a high optical responsivity of about 0.4 A W^−1^ in the wavelength range of 1260 to 1320 nm [27]. The photodetection wavelength of MoTe_2_ on a Si micro-ring has been extended to 1550 nm by using strain engineering [28]. Recently, Yang et al. demonstrated a MoTe_2_ photodetector on a proton-exchanged lithium niobate waveguide, achieving a high optical responsivity of 309 mA W^−1^ at 1310 nm and 13.8 mA W^−1^ at 1550 nm [29]. It should be noted that the integration of proton-exchange waveguides with other advanced LNOI photonic devices may present certain challenges [5].

On the other hand, the application of microresonators has recently made rapid progress in the fields of photonic devices, i.e., lasing, nonlinear photonics, and optical signal processing. Crucial properties of the resonators, such as the quality (Q) factor, can be affected by the structures and morphologies. For instance, Huang et al. prepared a ring resonator with a Q factor of 6.9 × 10^5^ on LNOI and compressed the linewidth of a 980 nm laser to 35 pm by self-injection locking [30]. Liu et al. achieved a 7.2 × 10^8^ Q factor and 380 μW threshold Brillouin lasing on silicon nitride waveguides [31]. Feng et al. combined electro-optic modulators with 6 × 10^6^ ring resonators on an LNOI for on-chip optical computations [32]. B. Desiatov et al. integrated the photodetector with a micro-ring resonator, having a Q factor of 1.5 × 10^5^, to realize Q-value detection in the visible light band [33]. As of now, there are relatively few research studies on the integration of the resonators into on-chip photodetectors on the LNOI platform.

In this paper, we report an on-chip photodetector in the telecommunication wavelength range based on 2D MoTe_2_ integrated with a lithium niobate waveguide and microresonator. The structures of LN waveguides and micro-ring resonators were fabricated by the femtosecond laser photolithography-assisted chemical–mechanical etching (PLACE) method. The dependence of the photodetector’s performance on the applied bias voltage, coupled-in light intensity and wavelength, and MoTe_2_ layer thickness is studied. The device features excellent performance metrics: a responsivity of 1 mA W^−1^ at the bias voltage of 20 V, a low dark current of 1.6 nA, and a photo-to-dark current ratio of 10^8^ W^−1^. The integration of the MoTe_2_ photodetector and micro-ring resonator on the thin film lithium niobate platform enables the measurement of the resonator’s quality factor, providing a promising scalable solution for the full realization of on-chip integrated photonic devices.

## 2. Materials and Methods

### 2.1. Materials and Fabrication of the On-Chip Photodetector

The few-layer MoTe_2_ was mechanically exfoliated from bulk crystals purchased from 2D Semiconductors Inc. (Scottsdale, AZ, USA). A commercially available X-cut LNOI wafer, fabricated by ion slicing (NANOLN, Jinan Jingzheng Electronics Co., Ltd., Jinan, China), was selected as the material for producing the LNOI waveguides in our experiment. The straight and micro-ring waveguide structures are fabricated based on the photolithography-assisted chemical–mechanical etching (PLACE) method [8] (more details are available in Section SI of Supporting Information). The Au electrodes on the sides of waveguides were deposited by magnetron sputtering and then the gap of 10 µm was removed by femtosecond laser ablation. Subsequently, few-layer 2H-MoTe_2_ flakes were exfoliated from bulk single crystals and transferred onto the electrodes and LN waveguide using the polydimethylsiloxane (PDMS) dry transfer method [34].

### 2.2. Mode Distribution and Absorption Simulation

Using Ansys Lumerical FDTD software (Ansys Lumerical 2024 R1), we simulated the mode distribution and energy intensity evolution of an LNOI waveguide integrated with 2H-MoTe_2_. The complex refractive index of 2H-MoTe_2_ at 1550 nm is n = 4.78 + 0.2i [35]. The designed structural parameters of the LN waveguide and the selective thicknesses of the MoTe_2_ were meticulously determined using atomic force microscope (AFM) characterization. Specifically, the waveguide structure was approximated to a trapezoidal shape, as vividly illustrated in the AFM images (Figure S1, Supplementary Materials). This structure encompasses a 290 nm thick slab layer and a 210 nm thick rib layer. The rib layer further comprises two symmetrical trapezoidal structures: the lower trapezoid with a base width of 7 μm, a top width of 2.5 μm, and a height of 140 nm, and the upper trapezoid with a base width of 1.2 μm, a top width of 1 μm, and a height of 70 nm. Additionally, a 13 nm thick layer of 2H-MoTe_2_ was incorporated. The absorption rate is characterized by η = 1–10^−0.1αL^, where L is the transmission distance and α is the mode absorption coefficient in dB μm^−1^. The thickness-dependent absorption was simulated by modifying the thickness parameter under the condition of the fundamental mode.

### 2.3. Experimental Setup for Q Factor of LN Micro-Ring Resonator

To evaluate the transmission spectrum and Q factor of the meticulously fabricated LN micro-ring resonator, a 1550 nm continuously tunable laser (CTL, TOPTICA Photonics Inc., Munich, Germany) is introduced into the chip through a lensed fiber (CXFIBER Inc., Wuhan, China). The output signal is then collected by the lens fiber and directed toward a commercial photodetector (New Focus 1811, Newport, Irvine, CA, USA). Subsequently, this signal is relayed to an oscilloscope (MSO 64, Tektronix Inc., Beaverton, OR, USA), enabling real-time monitoring of the resonant mode’s coupling status and facilitating precise measurements of the Q factor of the LN micro-ring. To optimize coupling efficiency, an in-line fiber polarization controller (PC) is utilized to adjust the laser’s polarization state. Furthermore, an arbitrary function generator (AFG3021C, Tektronix Inc., Beaverton, OR, USA) generates an external drive triangular wave electrical signal (100 Hz, 2 Vpp), which is applied to the CTL. This signal enables a fine wavelength scan centered around 1550 nm. Simultaneously, the electrical signal from the AFG is synchronized with the oscilloscope, ensuring accurate triggering and alignment of the transmission spectrum channel for detailed analysis.

### 2.4. Characterization of On-Chip Photodetector with Microresonator

The continuous-wave laser source was amplified using an erbium-doped fiber amplifier (EDFA) and efficiently coupled to the chip through a conical lensed fiber. A fiber-based polarization controller ensured that the polarization matched the required specifications. For the static photoresponse characterization of an on-chip photodetector, a Keithley 2400 source meter was used to apply a bias voltage and measure the resulting photocurrent. To characterize the photocurrent near the resonance wavelength of the fabricated LNOI micro-ring resonator, a tunable laser was coupled into the chip using a tapered lensed fiber, and the output was collected by the on-chip photodetector. Photocurrent curves were obtained by placing a probe on the chip detection electrodes and evaluating the static response using a semiconductor analyzer. Dark current measurements were conducted under various DC biases. For pulse response analysis, an intensity-modulated optical signal was directed onto the photodetectors, and the resulting electrical signal was captured and processed via an oscilloscope.

## 3. Results and Discussion

**Figure 1:**
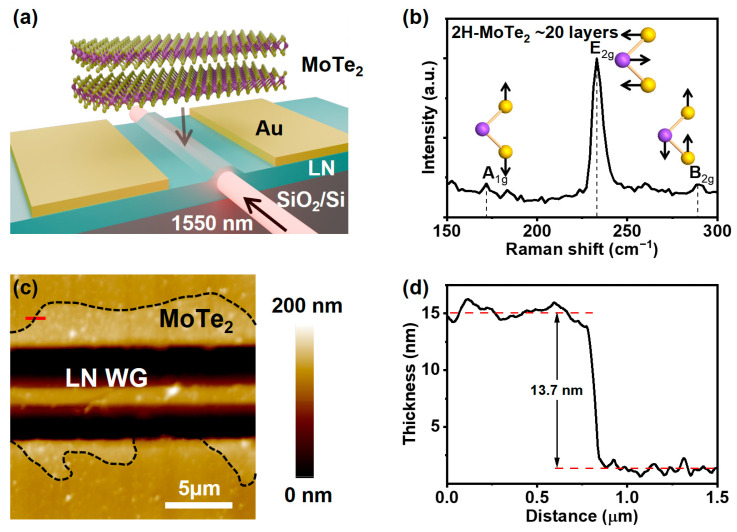
(**a**) Schematic diagram of the MoTe_2_-based on-chip photodetector. (**b**) Raman spectrum of 2H-MoTe_2_ (20 layers) on the LNOI platform under 532 nm laser excitation. Insert schematic of the MoTe_2_ structure: Mo (purple) and Te (yellow), the arrows indicate the direction of atom vibration. (**c**) AFM image of 2H-MoTe_2_ covering the LNOI waveguide (WG). (**d**) The thickness of the 2H-MoTe_2_ layer corresponds to the region of the solid line (red color) marked in (**c**).

Light with a wavelength of 1550 nm is coupled onto the LNOI waveguide, where it propagates in a largely confined manner and interacts with the 2H-MoTe_2_ layers. The light absorbed by the 2H-MoTe_2_ layers is subsequently converted into photocurrents, which are then collected by the electrodes. The schematic structure of the on-chip photodetector is shown in Figure 1a. It comprises a rib LNOI waveguide with Au electrodes positioned on both sides, with 2H-MoTe₂ bridging the gap between the electrodes and the waveguide. The LNOI waveguide is fabricated using the PLACE method. It features an upper width of 1 μm and a lower width of 7 μm, with an unetched depth of approximately 200 nm (more details are given in Figure S1, Supporting Information). The waveguide configuration is based on the parameters of previously demonstrated high-performance micro-ring resonator waveguides [30]. Subsequently, a 200 nm thin Au electrode with a gap of 7 µm was deposited at each side of the straight waveguides. Raman spectroscopy with an excitation laser wavelength of 532 nm was conducted to analyze the structure properties of 2H-MoTe_2_. The Raman single-point spectrum of transferred 2H-MoTe_2_ multilayers on LNOI is shown in Figure 1b. It is clearly observed that the three distinct peaks at 171.6, 233.3, and 289 cm^−1^ are consistent with the A_1g_ (out-of-plane), E_2g_ (in-plane), and B_2g_ (bulk inactive phonon) vibrational modes of 2H-MoTe_2_, respectively [36]. The morphology of the on-chip photodetector is revealed by atomic force microscopy (AFM) as shown in Figure 1c,d. It demonstrates homogenously flat 2D layers of 2H-MoTe_2_ after being transferred with the surface root mean square roughness of 4.5 nm. The coupling length between 2H-MoTe_2_ and the LNOI waveguide was measured to be approximately 20 μm. In addition, the thickness of 2H-MoTe_2_ was characterized as ~13.7 nm, corresponding to approximately 20 layers [37].

**Figure 2:**
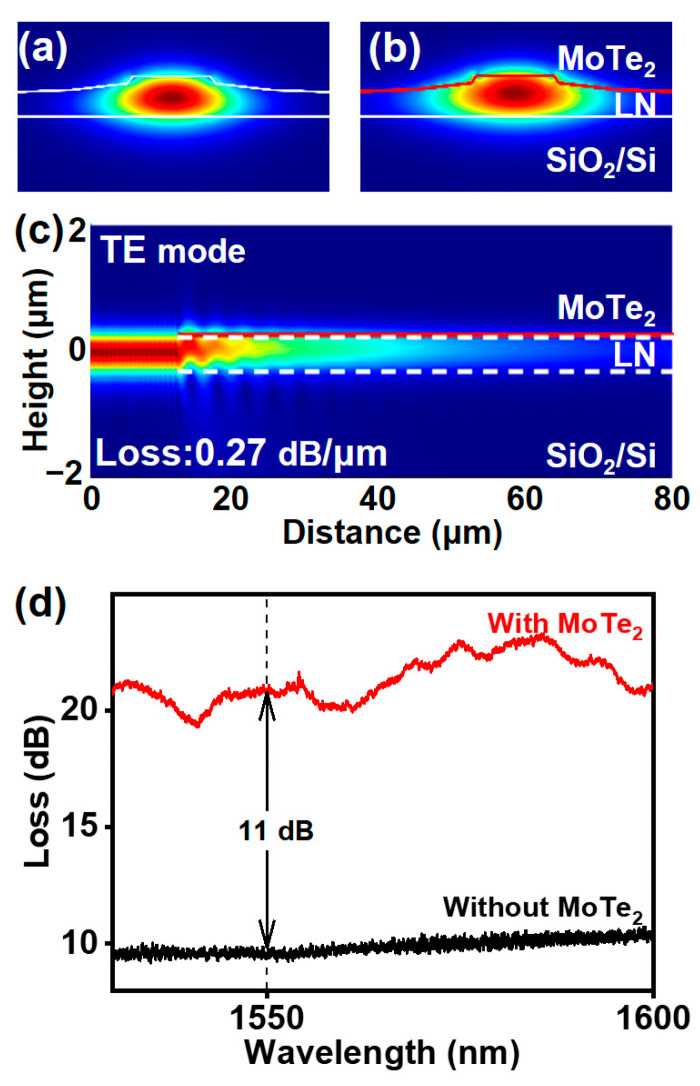
Simulation of the light field distribution in the LNOI waveguide without 2H-MoTe_2_ (**a**) and with 2H-MoTe_2_ (**b**). (**c**) The simulation of the electric field intensity |E^2^| in the coupling section for TE polarization. (**d**) Measured transmission loss of the waveguide without and with 2H-MoTe_2_.

To verify the on-chip photodetector design, we use the Finite-Difference Time-Domain method to simulate the optical field characteristics. The transverse electric (TE) mode fields of the LNOI waveguide without and with 2H-MoTe_2_ are illustrated in Figure 2a,b, respectively. The incorporation of 2H-MoTe_2_ modifies the mode field, causing an upward shift and broadening within the waveguide. Figure 2c shows the light field distribution along the propagation direction of the waveguide with 2H-MoTe_2_. Without considering the reflection and scattering, the transmission loss is 0.27 dB μm^−1^, which corresponds to a 72% absorptance rate of MoTe_2_ based on the absorption length of approximately 20 µm.

It should be noted that the simulated total device transmission loss of 5.7 dB is slightly lower than the experimental measurements, which are around 11 dB at 1550 nm. The experimental results were obtained by measuring the transmission loss of waveguides with 2H-MoTe_2_ and subtracting the loss of pure waveguides, including a fiber–waveguide coupling loss of approximately 10 dB, as shown in Figure 2d. Such an additional loss is possibly due to contamination during device fabrication [38].

**Figure 3:**
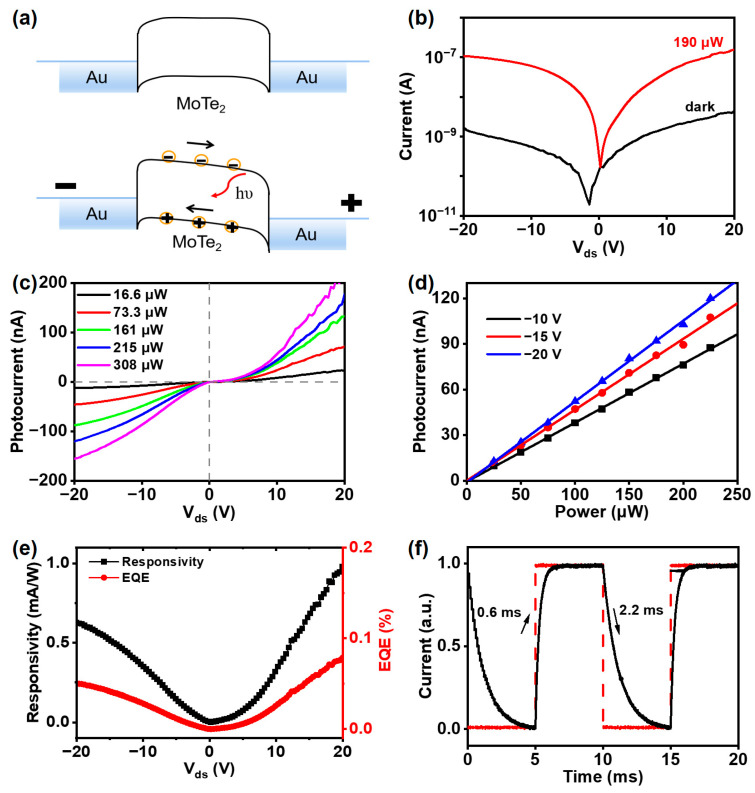
(**a**) Schematic band diagrams of the Au-2H-MoTe_2_-Au structure: (**top**) in thermal equilibrium; (**bottom**) under illumination and applied bias voltage. (**b**) Comparison of the I-V curves under dark and in-coupled light intensity with 190 μW. (**c**) I–V curves at varying light intensities. (**d**) Photocurrent as a function of light intensity within the waveguide at different bias voltages. (**e**) Responsivity and EQE as functions of bias voltage. (**f**) Impulse response curve of the photodetector.

The photocurrent generation mechanism of the photodetector is explained by the energy band diagrams as shown in Figure 3a. The photodetector at thermal equilibrium and with a positive bias is applied to the Au electrodes. A back-to-back Schottky junction, formed at the interface between the few-layer 2H-MoTe_2_ and the Au electrodes, acts as a barrier to carrier transport, minimizing dark current. When light of sufficient energy strikes the photodetector, photons are absorbed by the 2H-MoTe_2_ material, exciting electrons from the valence band to the conduction band and generating electron–hole pairs. The number of electron–hole pairs generated depends on the intensity of the incident light and the material’s optical absorption properties. Upon applying a bias voltage, the Schottky barrier is lowered, allowing electrons to be driven toward the positive electrode and holes toward the negative electrode. The external bias further enhances carrier separation, leading to the generation of photocurrent. Experimental results on I-V characteristics of the on-chip photodetector with and without coupled-in light are shown in Figure 3b. It is clearly observed that the photocurrent response is consistently two orders of magnitude higher than the dark current. Furthermore, due to the formation of a Schottky junction between 2H-MoTe_2_ and Au, the photodetector achieved a low dark current of 1.6 nA at a bias voltage of −20 V, which is seven orders of magnitude lower compared to that of the graphene-based on-chip photodetector (more details are given in Section SII and Figure S2, Supporting Information).

Derived from the I-V curves for the on-chip photodetector, photocurrent as a function of the applied bias voltage under various in-coupled light intensities is shown in Figure 3c. Here, the generated photocurrent is calculated by subtracting the dark current from the measured current with in-coupled light. The increase in bias voltage improves the carrier separation efficiency and enhances the photocurrent. Also, the photocurrent rises linearly with increasing light intensity from 16 to 300 μW, and without reaching saturation at these power levels. An important parameter of the on-chip photodetector that characterizes the efficiency of converting in-coupled light intensity (P_in_) into photocurrent (I_ph_) is responsivity, as expressed in the following equation [39]:

Responsivity = I_ph_/P_in_ (1)

Based on Equation (1) and the slope of the experiment data fitting, the responsivity of the on-chip photodetector can be extracted, and its behavior with various applied bias voltages is illustrated in Figure 3d,e. At a bias voltage of 20 V, the responsivity is determined to be 1 mA W^−1^ at 1550 nm, which corresponds to the external quantum efficiency (EQE) of 0.78% as given by

EQE = R × hc/qλ (2)

where R represents responsivity, h is Planck’s constant, c is the speed of light in vacuum, q is the fundamental electron charge, and λ denotes working wavelength [40]. The low EQE of the 2H-MoTe_2_-based on-chip photodetector is primarily due to the minimal absorption of 2H-MoTe_2_ at 1550 nm and the restriction of electron flow caused by the higher Schottky barrier. To measure the dynamic response of the photodetector, we modulated the in-coupled light with a frequency of 100 Hz, and the resulting curve is displayed in Figure 3f. The rise time (τ_rise) and fall time (τ_fall) are determined as the interval required for the photocurrent to escalate from 10% to 90% of its peak value and to decline from 90% to 10%, respectively. Consequently, the response speed of the photodetector was evaluated to be a rise time of 0.6 ms and a fall time of 2.2 ms.

**Figure 4:**
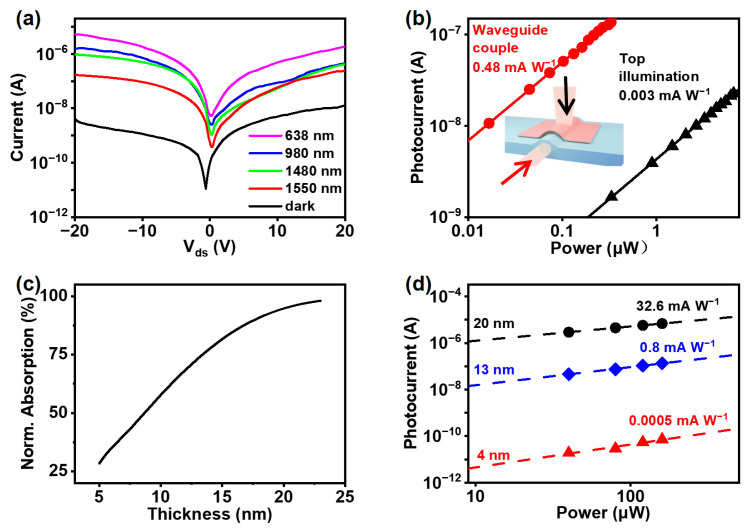
(**a**) I–V curves at a constant in-coupled light intensity of 300 μW with different wavelengths. (**b**) Comparison of the responsivity of the photodetector under in-coupled light via waveguide and spatial illumination. (**c**) Simulated absorption rate of 2H-MoTe_2_ at varying thicknesses. (**d**) 2H-MoTe_2_ thickness-dependent responsivity of on-chip photodetectors.

To examine the spectral response range of the 2H-MoTe_2_-based on-chip photodetector, we investigated the photocurrent response with varying wavelengths from 638 nm to 1550 nm while maintaining a constant in-coupled light intensity of 300 μW. Figure 4a presents the I–V characteristics of the photodetector as a function of the operating wavelength. The spectral photoresponse decreases as the irradiation light wavelength shifts from visible to infrared, reaching the microampere (µA) photocurrent order of magnitude at 1550 nm. Nonetheless, this indicates a broad spectral detection capability. Furthermore, Figure 4b compares the responsivity of the on-chip photodetector to light irradiation via top illumination. The input light intensity is calibrated, accounting for the waveguide coupling loss and the optical path loss for top illumination. Under a bias voltage of 10 V, the responsivity of the photodetector with the waveguide in coupled light is 0.48 mA W^−1^, which is 160 times higher than that of direct irradiation (0.003 mA W^−1^). This is because the waveguide in-coupled light with a transverse field enables efficient interaction with 2H-MoTe_2_ along the coupling length of 20 µm (in contrast to 13 nm thicker from top illumination) despite being weakly absorbed at 1550 nm.

Figure 4c,d show the influence of 2H-MoTe_2_ layer thicknesses on the performance of the on-chip photodetectors. As the thickness of 2H-MoTe_2_ increases, the simulated absorption rates increase almost linearly, approaching saturation at 90% when the thickness reaches 20 nm, as shown in Figure 4c. This can be attributed to the expanded overlap between the light field within the waveguide and the volume of 2H-MoTe_2_. These results are consistent with the measured responsivity-dependent 2H-MoTe_2_ thicknesses of the on-chip photodetector. The MoTe_2_ films with different thicknesses were obtained by combining the PDMS stamp-assisted mechanical exfoliation technique and AFM measurements. The photocurrent as a function of the in-coupled light power for the on-chip photodetector based on 2H-MoTe_2_ films with different thicknesses is presented in Figure 4d. The responsivity increases from 0.0005 to 32.6 mA/W as the 2H-MoTe_2_ layer thickness increases from 4 nm to 20 nm. This implies that it absorbs more light and generates more photocarriers. On the other hand, increasing the thickness of 2H-MoTe_2_ lowers the Schottky barrier height, thereby augmenting both the dark current and overall device responsivity [41]. The dark current and normalized photocurrent-to-dark current ratio (NPDR) for different thicknesses of 2H-MoTe_2_-based on-chip photodetectors were studied. It is shown that the dark current is as low as 10^−10^ A at a 4 nm thickness and the NPDR is as high as 10^8^ W^−1^ at a 13 nm thickness (more details are given in Section SIII and Figure S3a,b, Supporting Information). Our device exhibits an excellent performance of NPDR along with a significantly lower dark current, compared to that reported in the literature [28]. Such a high NPDR signifies a robust signal-to-noise ratio of the on-chip MoTe_2_ photodetector, which renders it highly advantageous for applications that require weak light detection. Despite the promising design of the integrated device, the experimental results reveal certain performance limitations, which can be attributed to the overall integration process. Specifically, the slightly lower performance of our device compared to previously reported devices is due to constraints imposed by the on-chip photonic integration process, the consistency of the waveguide structure, and the thinner thickness of the 2H-MoTe₂ layer.

**Figure 5:**
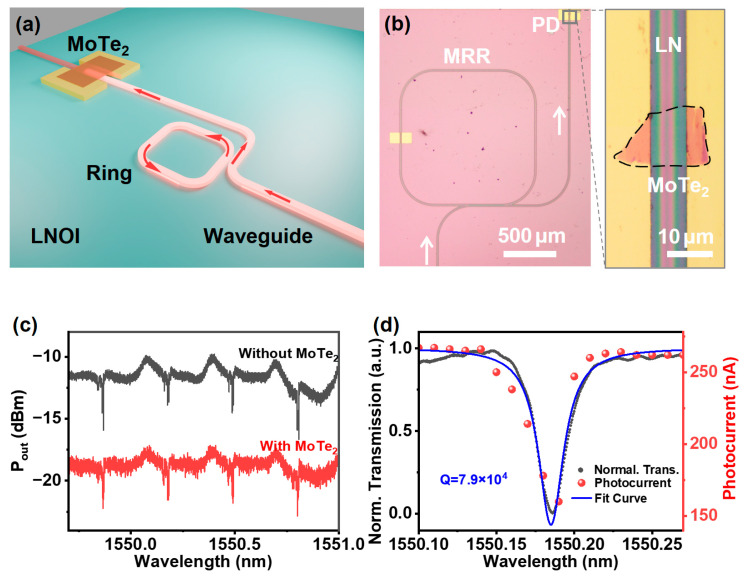
(**a**) Schematic diagram of the PD on a waveguide-coupled ring resonant cavity, the arrows indicate the direction of light propagation. (**b**) Optical micrograph of the micro-ring resonator (MRR) and the corresponding waveguide-integrated photodetector. (**c**) Output energy of the waveguide with and without 2H-MoTe_2_. (**d**) Comparison of transmission spectrum measured using commercial detectors and photocurrent measured using on-chip integrated PD, and Lorentz fitting curve.

We successfully integrated the 2H-MoTe_2_-based on-chip photodetector with a micro-ring resonator (MRR) on the same chip, as depicted in Figure 5a,b. The ring resonator adopted a box-like structure, wherein coupling between two straight waveguides was utilized instead of conventional ring structure coupling between a curved and a straight waveguide. This design increases the coupling length between the straight waveguide and the resonator, thereby enhancing the coupling efficiency. This approach was necessary due to the limitations imposed by the PLACE technology used for the LN waveguide fabrication, which restricts the coupling gap to approximately the micrometer range [31,42]. Light of a 1550 nm wavelength was launched into the LNOI waveguide and interacted with the MRR, before reaching the integrated 2H-MoTe_2_-based photodetectors. The transmission loss of the LNOI waveguide with and without 2H-MoTe_2_ was measured while tuning the wavelength around 1550 nm, as shown in Figure 5c. By comparing the transmission spectra with and without 2H-MoTe_2_, it is evident that the introduction of 2H-MoTe_2_ results in an approximate loss of 7 dB. This loss is attributed partly to light absorption by MoTe_2_ and the propagation process. The transmission spectrum shows an adjacent peak for each primary resonance, likely due to combining other modes as the LN waveguide supports TE_0_, TE_1_, and TM_0_ modes. Figure 5d shows the transmission spectrum of the MRR, which was characterized by the integrated 2H-MoTe_2_-based photodetector (red) and commercial photodetector (black). By fitting with a Lorentzian function, the quality factor of approximately 7.9 × 10⁴ for the fabricated ring resonator was extracted. The value of the Q factor is lower than the previously reported results owing to different ring structures and morphologies [30,31,32,33]. It would be expected to achieve a higher Q factor [8] when optimizing the PLACE manufacturing processes, improving the uniformity of the resonator structures, and reducing the coupling loss between the straight waveguide and the micro-ring structure. Nevertheless, the measured spectral photocurrent of the integrated photodetector closely matches that obtained from the commercial photodetector. Such wavelength-sensitive integrated photonic devices that combine an MRR filter and a 2H-MoTe₂-based photodetector suggest potential applications in the field of on-chip spectrometers and biosensors [43,44].

## 4. Conclusions

In summary, we successfully integrated the 2H-MoTe_2_ photodetector with a micro-ring resonator on the lithium niobate on the insulator platform, operating at the wavelength of 1550 nm. A responsivity of 1 mA W^−1^ was realized under a bias voltage of 20 V, which is about 160 times higher than that of free-space light irradiation. Due to MoTe_2_-Au forming Schottky junctions, the photodetector shows a low dark current of 1.6 nA and a reduction of approximately 10^7^ times as compared to the graphene-based photodetector. The integrated photodetector has a photo–dark current ratio of 10^8^ W^−^^1^. The demonstration of wavelength-selective detection via integration with a micro-ring resonator underscores the detector’s compatibility with various photonic devices. These findings indicate that the integration of MoTe_2_ photodetectors with microresonators on the LNOI platform is poised to play a pivotal role in the advancement of integrated lithium niobate photonics. Future efforts will focus on optimizing the coupling between the waveguides and MoTe_2_ and refining the micro-ring resonator design to further improve device performance and scalability.

## Data Availability

Data underlying the results presented in this paper are not publicly available at this time but may be obtained from the authors upon reasonable request.

## Supplementary Materials

The following supporting information can be downloaded at https://www.mdpi.com/article/10.3390/nano15010072/s1: Figure S1: AFM analysis of the cross-section of the fabricated LNOI waveguide. Figure S2: Dark current characteristics of 2H-MoTe_2_- and graphene-based on-chip photodetectors. Figure S3: (a) Dark current curves for photodetectors with varying thicknesses of 2H-MoTe_2_. (b) Normalized photocurrent–dark current ratio (NPDR) curves for photodetectors with different 2H-MoTe_2_ thicknesses.
