# Supplementary material for: MoTe2 Photodetector for Integrated Lithium Niobate Photonics
Source: Nanomaterials (Basel). 2025 Jan 5;15(1):72. doi: 10.3390/nano15010072 (PMC11723216; doi:10.3390/nano15010072)
Supplement: Supplementary file 1 [file nanomaterials-15-00072-s001.zip › nanomaterials-3404276-supplementary.pdf]

Supplementary Materials

$MoTe_2$ Photodetector for Integrated Lithium Niobate Photonics

Qiaonan Dong [1,2,3], Xinxing Sun [3,*], Lang Gao [1,3], Yong Zheng [3], Rongbo Wu [3] and Ya Cheng [1,3,*]

1 State Key Laboratory of High Field Laser Physics and CAS Center for Excellence in Ultra-Intense Laser Science, Shanghai Institute of Optics and Fine Mechanics (SIOM), Chinese Academy of Sciences (CAS), Shanghai 201800, China; dongqn@shanghaitech.edu.cn (Q.D.); langgao@siom.ac.cn (L.G.)

2 School of Physical Science and Technology, ShanghaiTech University, Shanghai 200031, China

3 The Extreme Optoelectromechanics Laboratory (XXL), School of Physics and Electronic Science, East China Normal University, Shanghai 200241, China; yong.zheng@stu.ecnu.edu.cn (Y.Z.); rbwu@phy.ecnu.edu.cn (R.W.)

* Correspondence: xxsun@phy.ecnu.edu.cn (X.S.); ya.cheng@siom.ac.cn (Y.C.)

Section SI: The photolithography-assisted chemical mechanical etching (PLACE) method

A commercially available Z-cut LNOI wafer was selected for our experiment to produce LNOI waveguides. The wafer consisted of a 500 nm-thick LN thin film bonded to a 2-μm-thick SiO_2 layer, which was grown on an Si substrate. The fabrication process began with the deposition of a 200 nm-thick chromium (Cr) layer on the LNOI surface using magnetron sputtering. This was followed by femtosecond laser ablation to pattern the Cr layer. To ensure the complete removal of the Cr without damaging the underlying LNOI, the laser pulse energy was carefully optimized, leveraging the unique material interaction properties of femtosecond laser pulses. A 100× objective lens with a numerical aperture (NA) of 0.7 was used to tightly focus the laser beam to a ~1 μm spot. The LNOI sample was translated using a computer-controlled XY motion stage, while the focus of the laser beam in the Z direction was controlled by another one-dimensional stage. A CCD camera mounted above the objective lens enabled real-time monitoring of the process. The patterned Cr layer served as a hard mask for the subsequent chemo-mechanical(CM) polishing step.

As the Cr film has a higher hardness than the LNOI, the polishing process selectively removed unprotected areas of LNOI, ensuring the material beneath the Cr mask remained intact. Following polishing, the Cr mask was removed by immersing the sample in a Cr etching solution for 30 minutes. For the fabrication of low-loss waveguides, an additional CM polishing step was performed at lower pressure and for a shorter duration to further enhance the smoothness of the LN waveguide's upper surface. The profile of the fabricated LNOI waveguides was characterized by AFM and the results are shown in Figure S1.

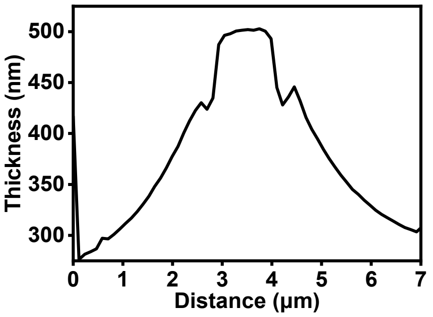

Figure S1. AFM analysis the cross-section of the fabricated LNOI waveguide.

Section SII: Fabrication of on-chip photodetector based on graphene on LNOI, comparison of the dark current between $MoTe_2$ and graphene-based on-chip photodetector

The graphene-based on-chip photodetector was fabricated by the same procedures as $MoTe_2$ photodetector in the main text. The dark current of the graphene-based photodetector is compared with that of a $MoTe_2$-based photodetector in Figure S2.

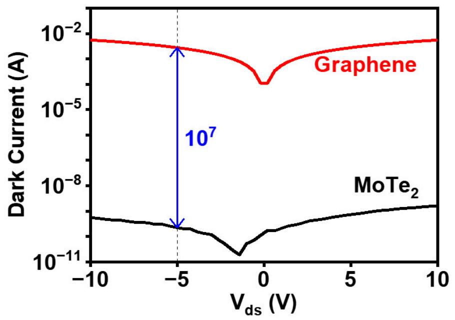

Figure S2. Dark current characteristics of 2H-$MoTe_2$ and Graphene based on-chip photodetector.

Section SIII: The thickness dependence of $MoTe_2$ on dark current and NPDR

The three difference layer thicknesses of $MoTe_2$-based on-chip photodetectors were fabricated. Their dark currents and normalized light-to-dark current ratios are investigated and shown in Figure S3.

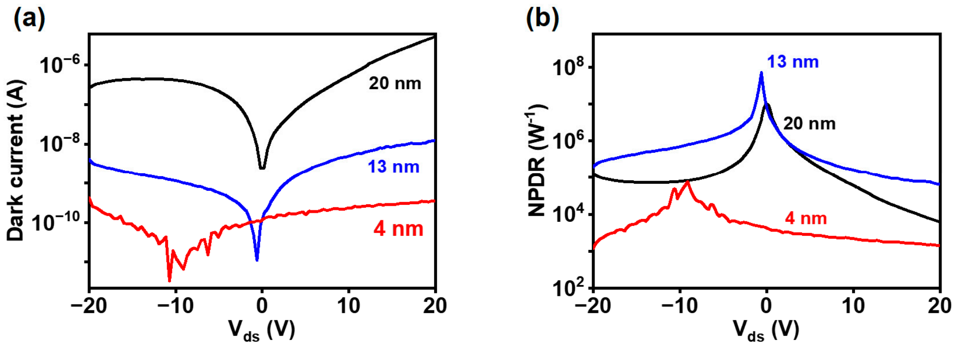

Figure S3. (a) Dark current curves for photodetectors with varying thicknesses of 2H-$MoTe_2$. (b) Normalized Photocurrent-Dark-Current Ratio (NPDR) curves for photodetectors with different 2H-$MoTe_2$ thicknesses.
